# TeaWeeding-Action: a vision-based dataset for weeding behavior recognition in tea plantations

**DOI:** 10.3389/fpls.2025.1722007

**Published:** 2025-12-16

**Authors:** Ru Han, Xinyi Liang, Lei Shu, Xiaoyuan Jing, Fan Yang, Renjie Tian

**Affiliations:** 1 Guangdong Provincial Key Laboratory for Green Agricultural Production and Intelligent Equipment, School of Computer Science, Guangdong University of Petrochemical Technology, Maoming, China; 2 College of Smart Agriculture (College of Artificial Intelligence), Nanjing Agricultural University, Nanjing, China; 3 School of Engineering and Physical Sciences, University of Lincoln, Lincoln, United Kingdom; 4 School of Electrical Engineering and Automation, Jiangsu Normal University, Xuzhou, China

**Keywords:** precision agriculture, weeding behavior recognition, tea plantation, computer vision dataset, object detection, smart farming

## Abstract

This study introduces a novel publicly available computer vision dataset specifically designed for weeding behavior analysis in tea plantations. Targeting the pressing challenges posed by weed infestations and aligning with global food security strategies, the dataset aims to advance intelligent weeding behavior recognition systems. The collection comprises 108 high-definition video sequences and 6,473 annotated images, capturing a wide range of weeding activities in real tea plantation environments. Data acquisition followed a hybrid approach combining field recordings with web-crawled resources, and encompasses six categories of weeding behaviors: manual weeding, tool-assisted weeding, machine-based weeding, tool-specific actions (including hoe and rake), handheld weeding machine use, and non-working states. A key innovation of the dataset lies in its multi-view acquisition strategy, integrating frontal, lateral, and top-down perspectives to ensure robust three-dimensional understanding of weeding behaviors. Annotations are provided in both COCO and YOLO formats, ensuring compatibility with mainstream object detection frameworks. Benchmark evaluations conducted with advanced algorithms such as YOLOv8, SSD, and Faster R-CNN demonstrate the effectiveness of the dataset, with Faster R-CNN achieving a mean Average Precision (mAP) of 82.24%. The proposed dataset establishes a valuable foundation for the development of intelligent weeding robots, precision agriculture monitoring systems, and computer vision applications in complex agricultural environments.

## 1 Introduction

Food security represents a fundamental strategic issue that directly affects national stability and livelihood. As the world’s most populous country, China considers agriculture not only as the cornerstone of its national economy but also as a crucial guarantee for long-term stability and social well-being. Nevertheless, global food security remains under severe pressure. According to the FAO (2024), approximately 733 million people worldwide faced hunger in 2023, with nearly 205 million individuals across 45 countries experiencing food insecurity at “crisis” or more severe levels Yubin et al. (2020); Su et al. (2024). These figures highlight the urgency of developing sustainable strategies to mitigate risks in agricultural production.

Among the multiple threats to food production, weed infestation poses a particularly persistent and complex challenge. Weeds compete with crops for light, nutrients, and space, disrupt normal growth, and serve as reservoirs for pests and pathogens Ren et al. (2015); Jin et al. (2021); Pai et al. (2024). In China, more than 85% of cultivated land suffers from weed damage, resulting in an estimated 10–15% yield loss annually, equivalent to over 60 million tons of grain and direct economic losses of approximately 120 billion RMB. Including ecological impacts, the total loss is estimated to exceed 220 billion RMB. With the added pressure of climate change and herbicide resistance, weed management has become a priority for ensuring food security.

Traditional weed control approaches—including manual weeding, mechanical removal, chemical herbicides, and mulching—each provide partial solutions but face clear limitations Dai et al. (2022). Manual weeding is labor-intensive and unsustainable under rural labor shortages; mechanical methods improve efficiency but leave gaps in coverage and demand high equipment costs; herbicides remain effective yet increasingly restricted due to ecological risks and resistance evolution; while mulching provides short-term benefits but contributes to plastic pollution in the long run. In this context, precision agriculture has emerged as a transformative paradigm, integrating GIS, RS, GPS, IoT, big data, and AI technologies to enable site-specific management, optimize resource use, and reduce environmental impacts Zhao et al. (2025).

Recent years have witnessed rapid progress in AI-driven intelligent weeding technologies, particularly robotic weeding systems. Major agricultural economies such as the United States, the European Union, and Japan have incorporated smart agriculture into national strategies, while China, although a late starter, has achieved remarkable advances in intelligent weeding robotics through domestic innovation and technology adaptation Sun et al. (2024); Yu et al. (2019). By 2024, autonomous weeding systems had been applied to diverse contexts including open fields, greenhouses, orchards, and tea plantations, reducing reliance on manual labor while enhancing operational standardization and efficiency.

The effectiveness of intelligent weeding robots critically depends on their ability to perceive complex agricultural environments and make adaptive decisions. Recent integration of geospatial knowledge graphs and AI has significantly improved environmental understanding, semantic mapping, and decision-making capacity, marking a paradigm shift in smart agriculture. Looking forward, emerging technologies such as 5G-A/6G communications, edge computing, large AI models, and digital twins will further expand the potential of intelligent weeding in sustainable agriculture.

Current agricultural vision datasets primarily target static plant or weed identification and lack the ability to model human–tool–behavior interactions in real weeding scenarios. Yet behavior perception is essential for next-generation intelligent weeding systems, which must distinguish operational states, identify tool usage, and interpret human actions under complex field conditions. Therefore, a dedicated, behavior-centered and multi-view dataset is necessary to support robust behavior recognition in agricultural environments.

Although robotic weeding systems aim to reduce manual labor, human weeding behaviors remain highly relevant for robotic perception and task execution. First, most agricultural robots currently operate in semi-autonomous settings, where humans and machines share the same workspace. Understanding human actions helps robots detect operator presence, recognize active working states, and maintain adaptive safety zones—capabilities widely emphasized in collaborative robotics research. Second, modeling human motion and tool-use patterns provides valuable priors for task allocation and behavior prediction, enabling robots to adjust their navigation or intervention strategies accordingly. Third, prior studies in agricultural HRI, imitation learning, and human intention recognition have demonstrated that recording human operational behaviors improves robotic decision-making and contextual awareness in field environments. Our dataset is therefore designed not to imitate manual weeding, but to support safer, more context-aware, and collaborative robotic operations in tea plantations.

Against this backdrop, our study focuses on constructing a novel computer vision dataset for tea plantation weeding behavior recognition. Unlike existing datasets in domains such as autonomous driving (e.g., COCO Han et al. (2025, 2024); Devi and Cn (2022), nuScenes Caesar et al. (2019)), object detection (e.g., VOC Ertler et al. (2019); Francies et al. (2021); Varadarajan et al. (2021); Shen et al. (2020)), and small-object behavior analysis (e.g., SM [16]), agricultural weeding datasets remain scarce, especially those addressing specific environments such as tea gardens. This study bridges this gap by developing a multi-perspective annotated dataset capturing six distinct weeding behaviors under real-world tea plantation scenarios. The dataset is provided in COCO and YOLO formats to ensure broad compatibility with state-of-the-art object detection frameworks. By establishing this resource, we aim to support advances in intelligent weeding robotics, precision agriculture, and food security.

Guide our dataset design, we propose the following hypotheses:

1. Multi-view imagery (frontal, lateral, top-down) provides complementary cues that significantly improve weeding behavior recognition.
2. Joint labeling of behavioral states and tool categories leads to more robust and discriminative feature learning.
3. Data collected from real tea plantation environments enhances model generalization in practical

## 2 Related works

### 2.1 Agricultural vision datasets

Although the demand for intelligent monitoring systems in agriculture has been rapidly increasing, publicly available datasets specifically designed for agricultural behavior recognition remain scarce. In particular, datasets targeting weeding behavior recognition are extremely limited, which hinders the development of robust deep learning models and real-world applications. Most existing agricultural vision datasets focus on crop disease identification, fruit detection, or static weed classification, such as PlantVillage, CropWeeds, and AgriVision Zhang et al. (2022); Lavrinenko (2023). While these datasets have proven effective for plant classification tasks, they primarily rely on static imagery, emphasizing plant or tool recognition without incorporating dynamic behavior, multi-view acquisition, or temporal context.

In contrast, large-scale general-purpose datasets such as COCO and Pascal VOC offer rich categories and standardized annotations but lack alignment with agricultural behavior scenarios. Their class definitions are not tailored to agricultural tasks—for instance, categories such as “hoe” or “handheld weeder” are absent, and behavioral states such as “manual weeding” or “tool-assisted weeding” are not defined. Consequently, models trained on these datasets often struggle to generalize effectively in real-world agricultural contexts.

To bridge this gap, the proposed TeaWeed dataset represents the first systematic attempt to capture and annotate diverse weeding behaviors in tea plantations. It integrates image and video data covering manual weeding, tool-based weeding, and machine-assisted weeding, while employing a multi-view acquisition strategy. By doing so, it provides richer spatial and behavioral features, enabling better generalization and robustness in complex agricultural environments.

### 2.2 Behavior recognition methods in agricultural scenarios

Behavior recognition in agricultural environments presents unique challenges, including complex backgrounds, occlusion, varying illumination, and action diversity. Traditional image processing techniques, such as color thresholding and edge detection, can be applied under controlled conditions but exhibit limited robustness under field conditions with fluctuating light and dense vegetation K. and Fredrik (2023).

In recent years, deep learning approaches have become the dominant paradigm. State-of-the-art object detection algorithms such as YOLO, Faster R-CNN, and SSD have been extensively applied to agricultural vision tasks, including crop counting, weed localization, and tool recognition. For example, Zhang et al. Zhang et al. (2022) employed an improved YOLOv5 model for potato sprout detection, while Sun et al. Zhang et al. (2024) introduced a Transformer-based multi-scale network for weed detection in sugarcane fields, demonstrating the potential of Transformer architectures in agricultural contexts.

Nevertheless, the majority of these studies focus on static object detection, such as crops, fruits, or weeds, rather than on dynamic behavior recognition. In weeding scenarios, most efforts have targeted the detection of tools or weeds themselves, without modeling the operator’s behavioral state. For example, one study on UAV-based weeding robots Kumar and Kant (2025) was able to detect the presence of weeds but could not determine whether a person was performing manual weeding, what tool was being used, or whether the operator was in a working or idle state.

To address these limitations, this study adopts a joint modeling strategy for behaviors and tools (joint modeling of behavioral states and tool-use patterns enables the system to understand not only the presence of humans but also their operational intentions, which is crucial for predicting task trajectories and avoiding interference, a point supported by recent works in agricultural HRI and collaborative robot perception) Li et al. (2020); Tung et al. (2024). Specifically, we introduce both behavioral state labels (manual weeding, tool-assisted weeding, non-working state) and tool category labels (hoe, rake, handheld weeder) to enable a comprehensive and fine-grained recognition of agricultural weeding activities. This labeling system not only supports conventional object detection tasks but also opens avenues for action recognition, temporal sequence modeling, and even evaluation of operation quality in agricultural workflows.

### 2.3 Data value and multi-view strategy

The key innovation of our dataset lies in its multi-view acquisition strategy, incorporating frontal, lateral, and top-down perspectives. This approach allows a more holistic capture of spatial and motion features of weeding behaviors, significantly enriching the feature representation for training models. Multi-view methods have already demonstrated their value in other computer vision fields. For instance, the TU-DAT dataset Santos and Gebler (2021) for traffic accident analysis employed multi-view CCTV footage to improve abnormal behavior detection, while Santos et al. Li et al. (2022) proposed a multi-view UAV-based apple detection system that combined CNN recognition with geometric modeling to overcome occlusion challenges. In the context of tea plantations, the multi-view approach addresses several critical issues:

- Occlusion: In dense vegetation, a single viewpoint often fails to capture tools or body movements completely; complementary perspectives mitigate missing information.
- Pose ambiguity: Frontal views facilitate tool-use detection, lateral views highlight motion direction, and top-down views separate ground vegetation from the working area.
- Background interference: Top-down imagery improves the model’s focus on behavior-relevant regions, reducing misclassification caused by cluttered backgrounds.

The proposed dataset includes finely annotated labels across six categories: manual weeding, tool weeding, machine weeding, tools (hoe, rake), handheld weeder, and non-working state. It incorporates multi-person, multi-session data, ensuring diversity and realism. To maximize usability, the dataset supports both COCO and YOLO formats, while preserving raw annotation files to facilitate conversion into other formats. This design enhances its compatibility with mainstream object detection frameworks and its scalability for future tasks.

## 3 Dataset description

Tea plantations present dense vegetation, frequent occlusion, and diverse tool-use patterns, making behavior recognition uniquely challenging. Focusing on six representative weeding behaviors within this environment allows the dataset to align closely with the perceptual needs of intelligent weeding systems and provides a meaningful benchmark for agricultural behavior analysis.

**Figure 1:**
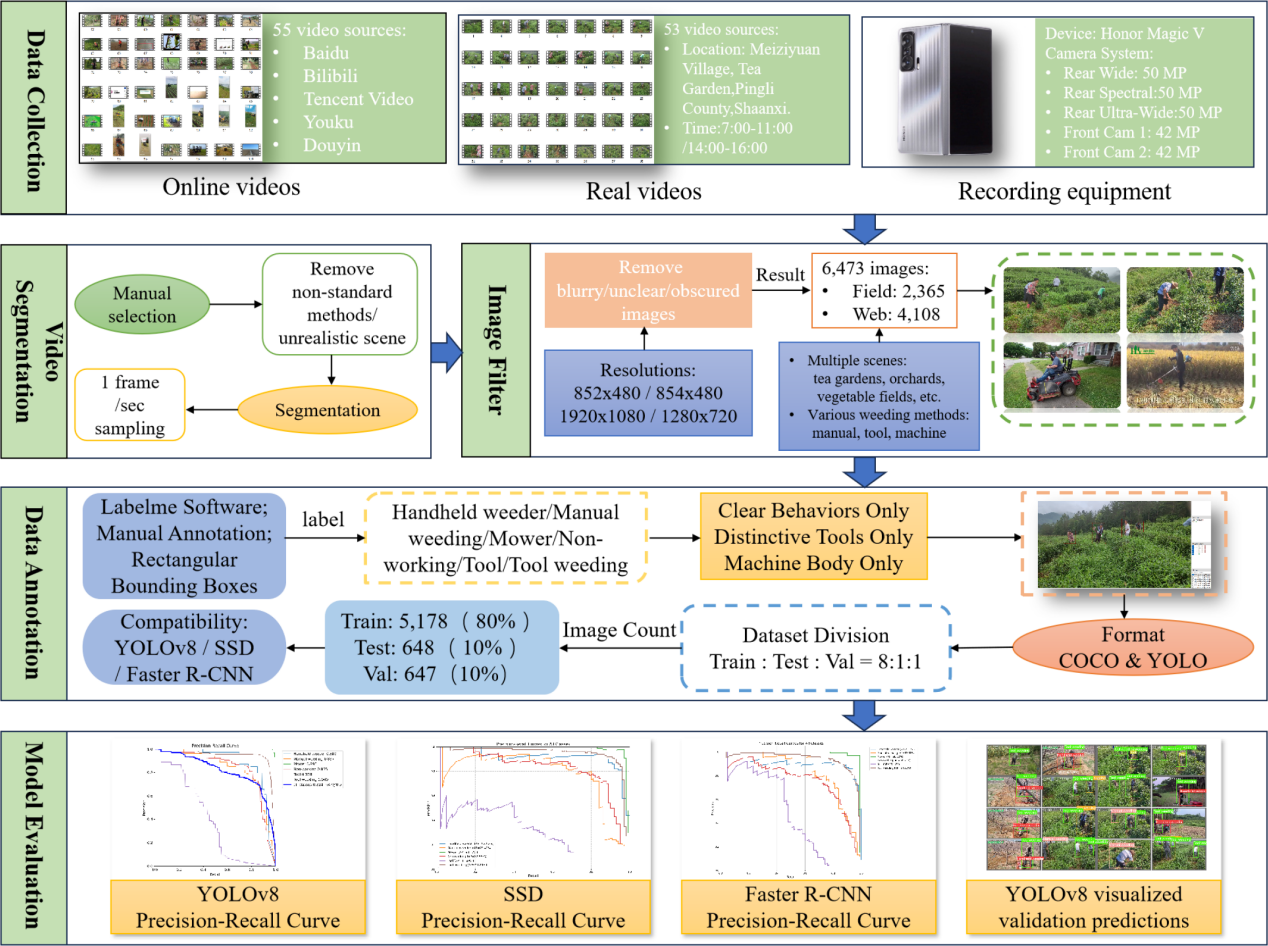
Training curves of baseline - extended models: performance evolution with architectural add – ons.

In this study, we systematically constructed a high-quality open dataset for weeding behavior recognition in tea plantations, with the complete figure illustrated in **Figure 1**. The figure outlines a closed-loop workflow that spans from data collection, video slicing, and image preprocessing to annotation and model evaluation, thereby providing a comprehensive overview of the dataset construction process.

The upper section of **Figure 1** highlights the two primary sources of data acquisition: (i) web crawling from mainstream platforms such as Baidu, Bilibili, and Tencent Video, ensuring broad coverage of real-world agricultural activities; and (ii) on-site filming conducted in tea plantations located in Meiyuan Village, Pingli County, Ankang City, Shanxi Province, which guarantees contextual authenticity and environmental diversity.

The middle section of the figure illustrates the core steps of video processing, including rigorous video selection, slicing, and image preprocessing procedures, which were implemented to standardize the raw input data and enhance image quality.

The lower section presents the annotation and validation phases. Fine-grained manual labeling was performed using the Labelme tool, covering six categories of weeding behaviors and tools. Subsequently, the dataset’s utility and reliability were validated through extensive experiments with state-of-the-art algorithms, including YOLOv8, SSD, and Faster R-CNN, all of which demonstrated the effectiveness of the dataset for training and benchmarking agricultural behavior recognition models.

Overall, the construction workflow not only ensures the diversity, representativeness, and technical rigor of the dataset but also provides a generalizable methodological reference for future dataset development in complex agricultural environments.

### 3.1 Dataset construction

The construction of the dataset can be divided into four core stages: data acquisition, video screening and slicing, image annotation, and dataset partitioning.

#### 3.1.1 Data acquisition

The image sources of this dataset are derived from 108 high-definition video resources. Among them, 55 videos were collected via web crawling from multiple online platforms, including Baidu, Bilibili, Tencent Video, Youku, and Douyin, ensuring diverse sources and rich tea plantation scenarios. The remaining 53 videos were captured on-site in the Meziyuan Village, Dangmenpo Tea Garden, Pingli County, Ankang City, Shaanxi Province. Filming was conducted during morning (7:00–11:00) and afternoon (14:00–16:00) periods to capture operational scenes under varying illumination conditions. The on-site recordings were performed using the Honor Magic V foldable smartphone, equipped with a rear triple camera of 50 MP and a front dual camera of 42 MP, providing high-resolution and detailed raw material.

**Figure 2:**
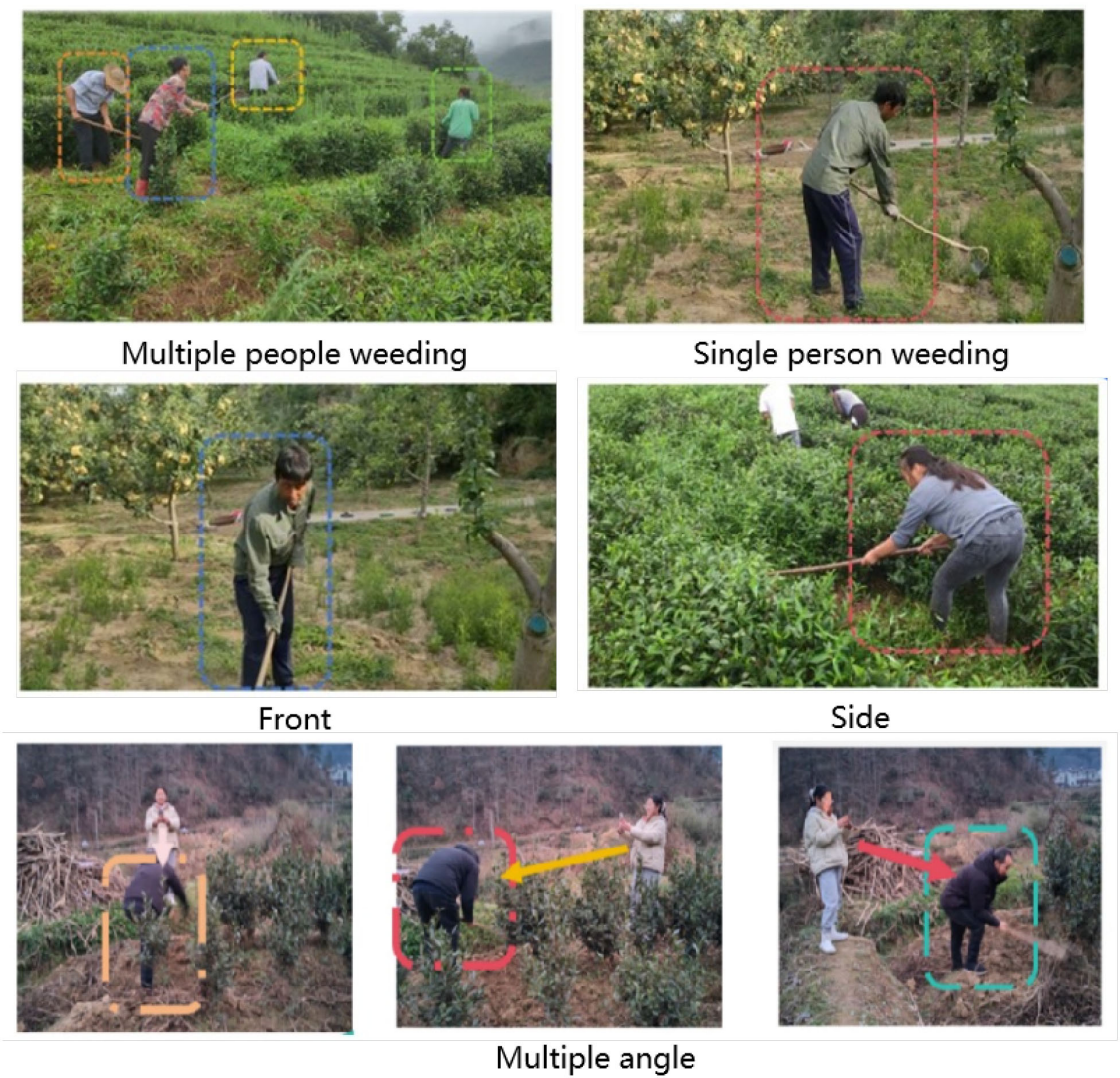
Illustration of multi-person, multi-view, and multi-angle scenarios in agricultural weeding activities.

A novel aspect of this dataset is the adoption of three standard perspectives—frontal, lateral, and top-down—for image acquisition, as illustrated in **Figure 2**. The frontal view facilitates accurate recognition of facial expressions and tool usage, offering key features for behavioral assessment. The lateral view captures human motion direction and spatial relationships more clearly, mitigating posture ambiguity. The top-down view provides comprehensive spatial layout information, separating ground vegetation from operational areas, and significantly reducing background interference. This multi-perspective acquisition strategy enables models to learn three-dimensional spatial perception and behavioral understanding effectively, addressing common challenges associated with occlusion, posture ambiguity, and background confusion in single-view datasets.

Additionally, the dataset encompasses both single-person operations and collaborative multi-person scenarios, demonstrating coverage of complex real-world conditions. All acquired videos were manually screened to retain only compliant operational behaviors, while excluding exaggerated or non-standard practices. The dataset includes three weeding modalities: manual hand weeding, tool-assisted weeding, and mechanical weeding. Tool-assisted weeding further covers three types of implements: hoes, rakes, and handheld weeders. The varying tea plantation backgrounds, vegetation densities, and ground textures provide diverse environmental conditions, offering robust training samples for model generalization. Original video resolutions range from 1080P (1920×1080) and 720P (1280×720) to 480P (852×480/854×480).

#### 3.1.2 Video screening and slicing

To efficiently extract key frames, all videos were sliced at a fixed interval of one frame per second. Considering the heterogeneous quality of web-crawled videos, some slices might not contain relevant labels as described in Section 3.1.1. Such frames could potentially introduce noise and degrade the training performance. Therefore, the slicing process was combined with a rigorous two-step manual screening procedure. While ensuring continuity of retained video segments, non-compliant frames were removed according to strict criteria, including: (i) blurred images, (ii) ambiguous behaviors, (iii) occlusion of the weeding subject by vegetation or other objects, and (iv) incomplete visibility of the entire weeding action.

Following this pipeline, a total of 6,473 images were obtained, among which 2,365 originated from field recordings and 4,108 from online videos, thereby enriching both scene diversity and behavioral variability. The resulting image resolutions primarily included 852×480, 854×480, 1280×720, and 1920×1080, ensuring compatibility with diverse model input requirements. Moreover, the naming convention preserved the temporal sequence of images extracted from each video, providing a structured foundation for potential future temporal action analysis.

#### 3.1.3 Data annotation

Image annotation represents a critical step to ensure dataset quality. In this study, we adopted Labelme, a widely used open-source annotation tool, to achieve precise and consistent labeling. Six behavior categories were defined in the dataset: (i) manual weeding; (ii) tool weeding; (iii) mower (mechanical weeding); (iv) tool (hoe/rake); (v) handheld weeder; (vi) non-working. Among these, four categories—manual weeding, tool weeding, mower, and non-working—describe the behavioral states of human subjects, whereas tool (hoe and rake) and handheld weeder correspond to specific tool types.

In this dataset, human behaviors and tools are treated as two distinct annotation categories. Behavioral classes include: manual weeding, tool weeding, machine weeding, and non-working state. Tool classes (hoe, rake, handheld weeder) are annotated separately as object categories. Although tools frequently appear within tool-weeding behaviors, they are not part of the behavior labels themselves. This separation avoids conceptual overlap and allows independent evaluation of (1) human behavioral state recognition and (2) tool object detection.

All targets were annotated using rectangular bounding boxes, which were required to tightly enclose the object without excessive background inclusion or loss of critical details. To ensure annotation reliability, we calculated inter-annotator agreement using Cohen’s Kappa. The resulting score was *κ* = 0.78-0.85, indicating substantial agreement among annotators. Annotation adhered to strict principles:

- Only targets with clearly identifiable behavioral states were annotated.
- Subjects with incomplete actions, ambiguous intentions, or transitional states were excluded.
- Human or machine subjects were annotated only if largely visible within the frame; partially visible limbs or fragmented machine parts were excluded.
- Distant or excessively small targets with blurred features were excluded.
- Only tools with distinct and classifiable appearances (e.g., hoe, rake, handheld weeder) were annotated, while heavily occluded, indistinct, or unclassifiable objects were ignored.
- For mechanical weeding, only the machine itself was annotated, and operators of remote-controlled devices were not labeled.

**Figure 3:**
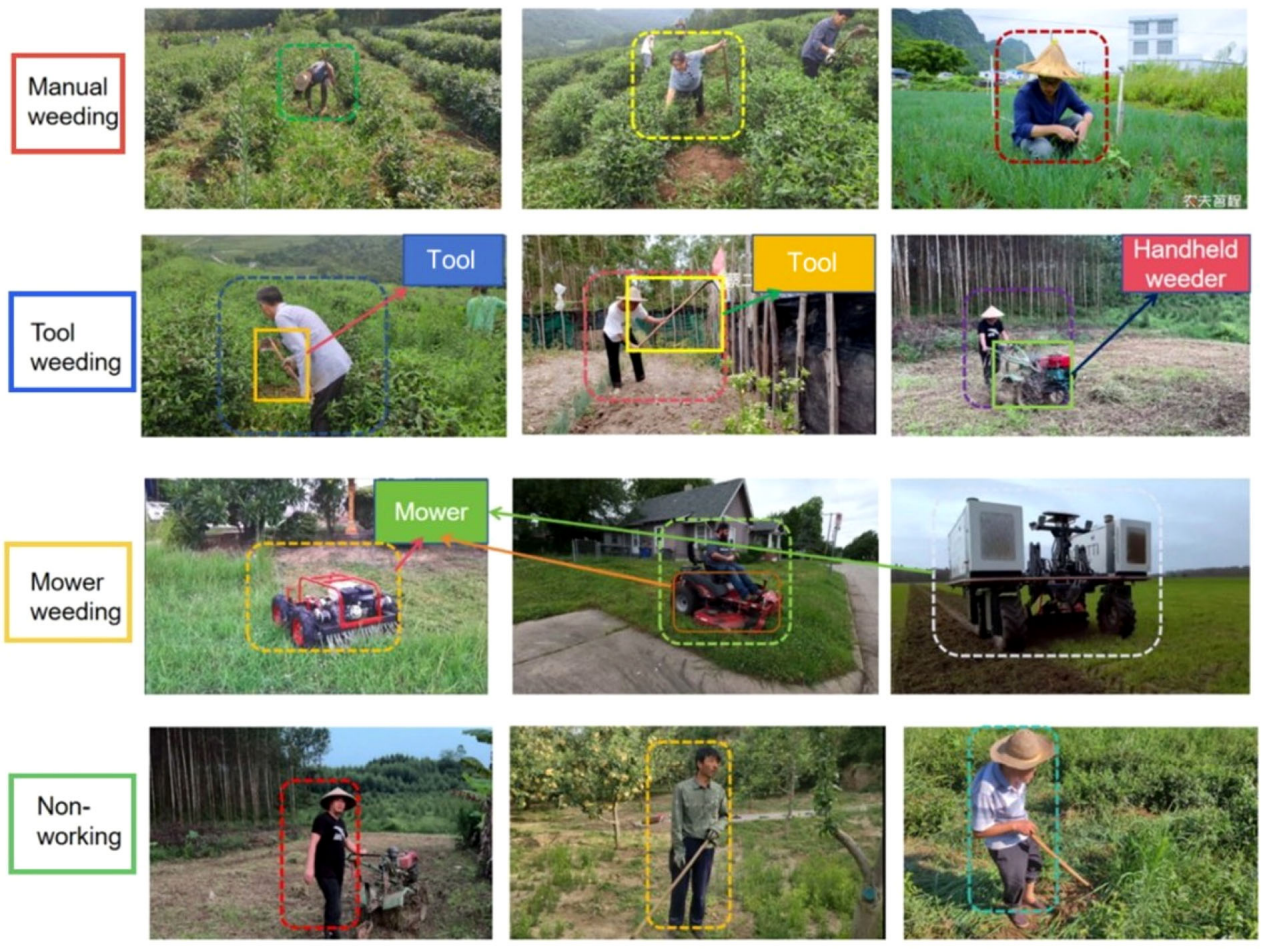
Illustration of the dataset annotation situation.

Representative annotation examples are shown in **Figure 3**, which illustrates four annotated states across diverse tea plantation scenarios. Within the tool-weeding category, three subtypes—rake, hoe, and handheld weeder—are explicitly demonstrated under different temporal conditions, including morning, noon, and evening.

In addition, **Table 1** presents the core dimensional characteristics of the dataset. It not only provides a clear breakdown of the six behavioral categories with their corresponding numbers of scenes and images but also further categorizes the data according to the number of participants (i.e., single-person operations and multi-person collaborations) and shooting distances (i.e., close-range, medium-range, and long-range). By consulting this table, researchers can rapidly grasp the dataset’s structural properties in terms of label distribution, scene diversity, participant configurations, and viewpoint variability. Such a systematic summary enables a comprehensive and transparent understanding of the dataset composition.

**Table 1:** Statistics of the dataset, including label distribution, number of scenes, and image counts across different categories.

Label		Scenes	Images
Manual weeding		3	932
Tool weeding	Hoe	2	155
	Rake	3	709
Handheld lawn mower		4	597
Machine weeding		3	1536
Non-working state		3	1451
Number of people	Single people	7	2170
	Multiple people	4	2780
Distance	Long-distance	5	2611
	Close-distance	4	2916
	Both	2	946

#### 3.1.4 Dataset division

Considering the wide application of the YOLO family of models (e.g., YOLOv5, YOLOv8, YOLOv9) in object detection and their requirement for specific annotation formats, all original JSON annotations generated by Labelme were batch-converted into YOLO format using custom Python scripts. Meanwhile, the original Labelme JSON files were fully preserved to ensure both dataset integrity and future extensibility.

Subsequently, the 6,473 annotated images together with their corresponding annotation files (in both COCO and YOLO formats) were randomly shuffled and split into three subsets following an 8:1:1 ratio: 5,178 images (80%) for training, 647 images (10%) for validation, and 648 images (10%) for testing. The dataset thus provides multiple annotation formats, facilitating compatibility with a broad range of object detection frameworks while ensuring reproducibility and scalability.

## 4 Methods

### 4.1 Evaluation framework

To validate the effectiveness and quality of the proposed tea plantation weeding behavior recognition dataset in model training, we adopted three state-of-the-art object detection algorithms: YOLOv8, SSD, and Faster R-CNN. These models represent different architectural paradigms in object detection, thereby enabling a comprehensive evaluation of the dataset across multiple methodological perspectives. We adopted official default hyperparameters to ensure fair and reproducible benchmarking, and did not perform cross-validation because model optimization is not the focus of this dataset-oriented study.

1. YOLOv8 represents the latest evolution of the YOLO family, characterized by its single-stage architecture and superior real-time detection capability.
2. SSD (Single Shot MultiBox Detector) provides another single-stage approach that leverages multi-scale feature maps to detect objects of varying sizes.
3. Faster R-CNN exemplifies a two-stage detector, which first generates region proposals and subsequently classifies them, generally achieving higher accuracy at the expense of computational efficiency.

### 4.2 Experimental settings

The experiments were conducted under the following configuration: the operating system was Ubuntu 20.04, equipped with an NVIDIA GeForce RTX 2080 Ti GPU, and implemented using PyTorch 2.0. To ensure fair comparison, all models were trained, validated, and tested on the same dataset partitions, with identical preprocessing pipelines applied.

During training, the batch size was set to 16, the initial learning rate to 0.001, and the models were trained for 100 epochs. These consistent settings ensured the comparability of results across different algorithms.

### 4.3 Evaluation metrics

The performance of the object detection algorithms was assessed using standard metrics in computer vision, The following relevant index equations can be found in Equations 1–4:

• Precision (P): the ratio of correctly predicted positive instances to the total predicted positive instances, defined as

(1) $$\text{Precision}=\frac{TP}{TP+FP}$$

where TP denotes true positives and FP denotes false positives.

• Recall (R): the ratio of correctly predicted positive instances to all actual positive instances, defined as

(2) $$\text{Recall}=\frac{TP}{TP+FN}$$

where FN denotes false negatives.

• F1 Score: the harmonic mean of precision and recall, providing a balanced measure of both, defined as

(3) $$F1=2\times \frac{\text{Precision}\times \text{Recall}}{\text{Precision}+\text{Recall}}$$

• Mean Average Precision (mAP): the mean of average precision scores across all categories, computed as the area under the precision–recall curve for each class, then averaged across all classes. Formally, for N classes:

(4) $$mAP=\frac{1}{N}\sum_{i=1}^{N}AP_{i}$$

where *AP_i_* denotes the average precision of the *i^th^* class. These metrics collectively provide a rigorous and comprehensive evaluation of detection performance, enabling detailed analysis of strengths and limitations across different weeding behaviors and environmental conditions.

In this study, we adopt mAP@0.5 as the primary evaluation metric, as it is widely used for baseline benchmarking in object detection tasks and aligns with our goal of providing a foundational dataset for weeding behavior recognition without introducing excessive complexity.

## 5 Dataset verification

### 5.1 Overall performance comparison

To comprehensively evaluate the effectiveness of the proposed tea plantation weeding behavior recognition dataset, we conducted extensive experiments using three representative object detection algorithms: YOLOv8, SSD, and Faster R-CNN. These models encompass both single-stage and two-stage detection paradigms, enabling a holistic assessment of dataset quality in terms of detection accuracy, robustness, and adaptability to complex field scenarios. The results provide insights into how different architectural designs handle variations in object size, background complexity, and operational conditions.

The overall performance of these algorithms on the dataset is summarized in **Table 2**. As shown, Faster R-CNN achieved the highest mean Average Precision (mAP) of 82.24%, indicating superior overall detection capability in tea plantation weeding scenarios. YOLOv8 exhibited the highest Precision at 83.7%, suggesting fewer false positives in its predictions. SSD demonstrated relatively balanced performance across all metrics, although its overall performance was slightly lower than the other two models.

**Table 2:** Comparison of detection performance across different object detection models on the target dataset.

Model	Precision (%)	Recall (%)	mAP (%)	F1 score (%)
YOLOv8	83.7	79.2	81.4	81.4
SSD	78.5	76.3	79.88	77.4
Faster R-CNN	80.1	81.5	82.24	80.8

**Figure 4:**
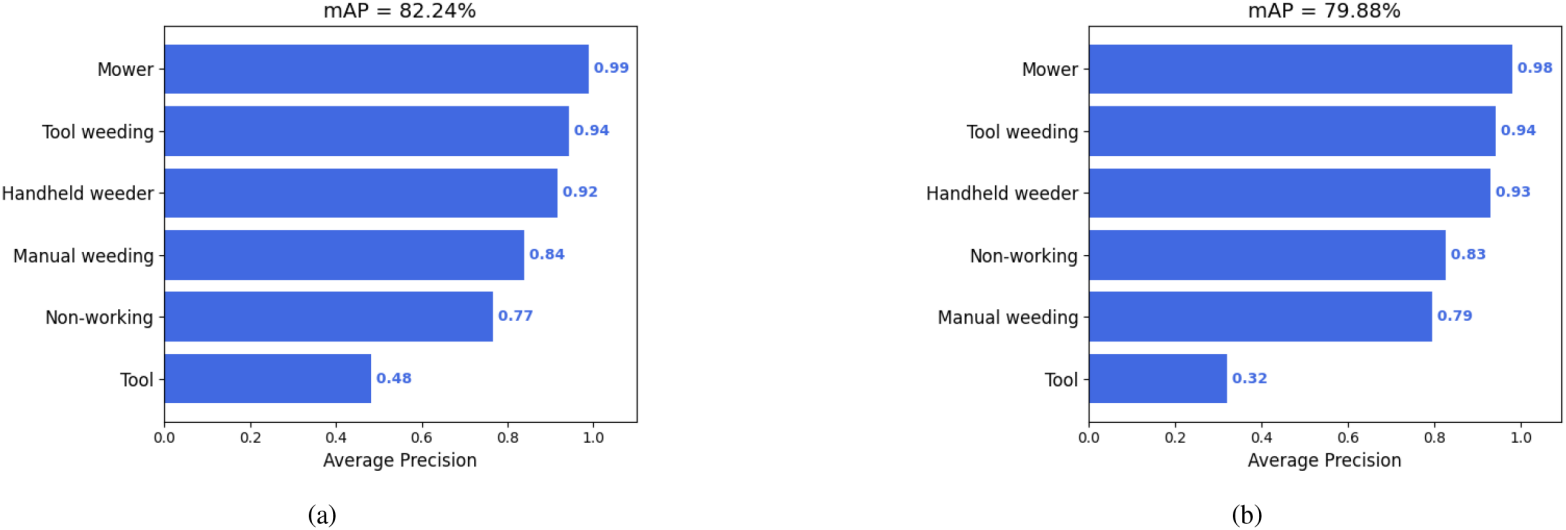
**(a)** Average precision (AP) per class for the Faster R-CNN model, achieving an overall mAP of 82.24%, with strong performance on most categories except “Tool”. **(b)** AP per class for the SSD model, showing slightly lower mAP of 79.88% and reduced performance on the “Tool” class compared to Faster R-CNN.

In addition, **Figure 4** illustrates the average precision (AP) distributions for Faster R-CNN and SSD, allowing detailed comparison of their detection behavior across different classes. Faster R-CNN achieved an mAP of 82.24%, whereas SSD reached 79.88%, resulting in a 2.36-percentage point difference. This indicates that Faster R-CNN provides more precise localization and classification of weeding behaviors, particularly in challenging conditions such as complex backgrounds and small objects (e.g., weeding tools). In contrast, SSD, as a single-stage detector, benefits from multi-scale feature maps to improve detection speed, but its feature extraction precision is inherently limited. This limitation leads to higher susceptibility to missed or incorrect detections for small-scale targets (e.g., distant tools) and dense scenes (e.g., multi-person weeding operations).

Overall, these results highlight the trade-offs between accuracy and computational efficiency across different detection architectures, while also demonstrating the dataset’s capability to support robust training and evaluation of diverse object detection models in realistic agricultural scenarios.

### 5.2 Category-wise detection performance analysis

**Figure 5:**
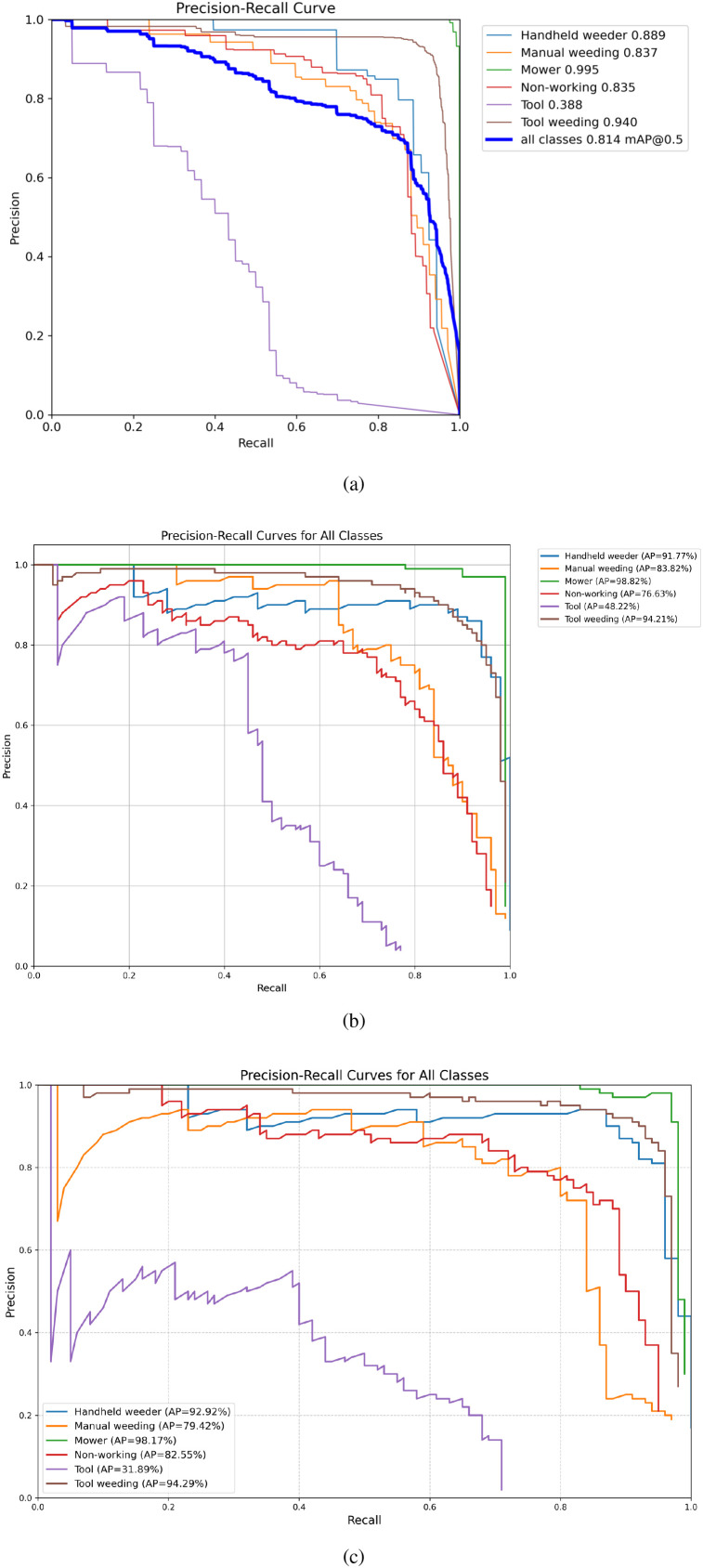
**(a)** Precision-recall curve for YOLOv8 on the target dataset, showing high precision and recall across all classes with an overall mAP@0.5 of 0.814. **(b)** PR curve for Faster R-CNN, demonstrating competitive performance with strong recall but slightly lower precision for certain classes. **(c)** PR curve for SSD, exhibiting rapid drop in precision at higher recall values, indicating challenges in handling dense or small-scale objects.

The detection performance of the three models was evaluated using Precision-Recall (PR) curves and Average Precision (AP) values for each weeding behavior category, as illustrated in **Figure 5**. A comparative analysis reveals distinct performance trends across categories:

1. Mechanical Weeding (Mower): All three models achieved high detection accuracy for this category, with Faster R-CNN and YOLOv8 yielding AP values close to 1.0. This indicates that mechanical weeding targets exhibit highly distinctive visual features, making them relatively easy to detect. The superior performance can be attributed to the large size and unique shape of the mowing machines, which stand out clearly in the image data.
2. Handheld Weeder: YOLOv8 demonstrated the best performance for this category, achieving an AP of 0.889, followed by Faster R-CNN, while SSD lagged behind. This can be explained by YOLOv8’s architecture, which is well-suited for detecting medium-sized objects. Handheld weeders typically fall within this size range, enabling YOLOv8 to capture multi-scale features more effectively.
3. Manual Weeding: YOLOv8 again outperformed the other two models, achieving an AP of 0.837. Manual weeding involves significant variation in posture and movement, leading to diverse visual appearances. YOLOv8’s multi-scale feature extraction and enhanced representation capacity allow it to robustly handle the variability inherent in manual weeding behaviors.
4. Tools: Detection performance for tools was relatively poor across all models, particularly for SSD and Faster R-CNN. This is primarily due to the limited number of annotated samples for this category and the typically small size and frequent occlusion of tools in the images. Moreover, the tools category encompasses multiple subclasses, such as hoes and rakes, which further increases intra-class variability and complicates the detection task.

From the PR curve analysis, additional observations can be made regarding model stability and robustness:YOLOv8 consistently maintains higher precision across varying recall levels, demonstrating smooth degradation in precision even at high recall values, which indicates robust detection performance under challenging scenarios. Faster R-CNN excels in high-precision regions but exhibits a steep decline in precision as recall increases, suggesting a higher likelihood of false positives when prioritizing recall. In contrast, SSD shows generally lower performance, with pronounced precision drops at higher recall levels, highlighting its relatively weaker stability in complex field conditions.

Overall, these results indicate that YOLOv8 achieves a favorable balance between precision and recall across all weeding behavior categories, making it the most suitable model for real-world tea garden weeding detection applications.

**Figure 6:**
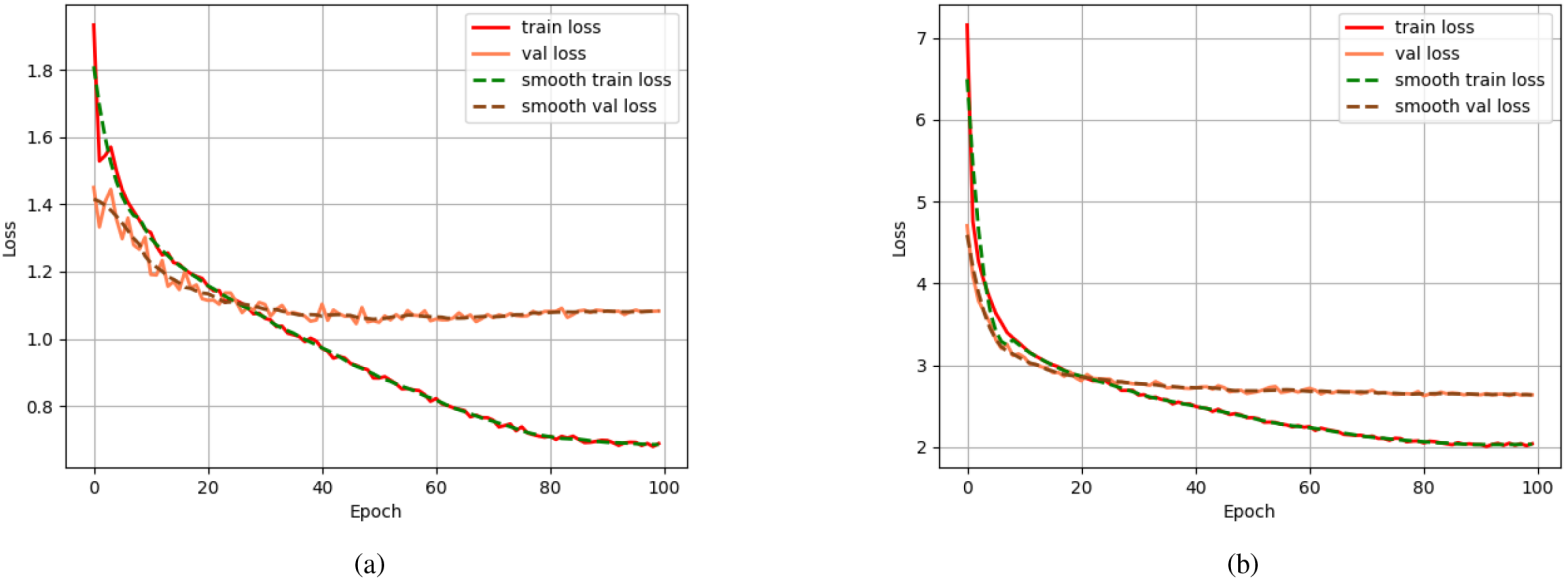
This is training and validation loss curves over epochs for two object detection models.**(a)** Loss dynamics of the Fast R-CNN model, showing convergence behavior of training (red) and validation (orange) losses, with smoothed trends overlaid. **(b)** Corresponding loss curves for the SSD model under the same training conditions, exhibiting faster initial convergence but higher final validation loss compared to Fast R-CNN. Schemes follow the same formatting.

In the context of tea garden weeding detection model validation, five key figures analyzing training and validation losses for Faster R-CNN, SSD, and YOLOv8 are systematically examined. For Faster R-CNN (**Figure 6a**), both training (red solid line) and validation (brown dashed line) losses initiate at high values (7.0 and 6.5, respectively) and exhibit rapid early decline, stabilizing by epoch 100 at 3.0 (train) and 2.0 (val). Their smoothed counterparts (green/blue dashed lines) reinforce consistent convergence without significant fluctuations, indicating stable generalization and minimal overfitting as training and validation losses approach asymptotic minima. In contrast, SSD (**Figure 6b**) displays a pronounced divergence: training loss (red solid) plummets from 1.8 to 0.7–0.8 by epoch 100, while validation loss (orange solid) plateaus early at 1.1–1.2 post-epoch 20. This widening gap between smoothly tracked series (green/brown dashed) signals overfitting, where the model optimizes excessively for training data but fails to generalize to unseen validation samples.

**Figure 7:**
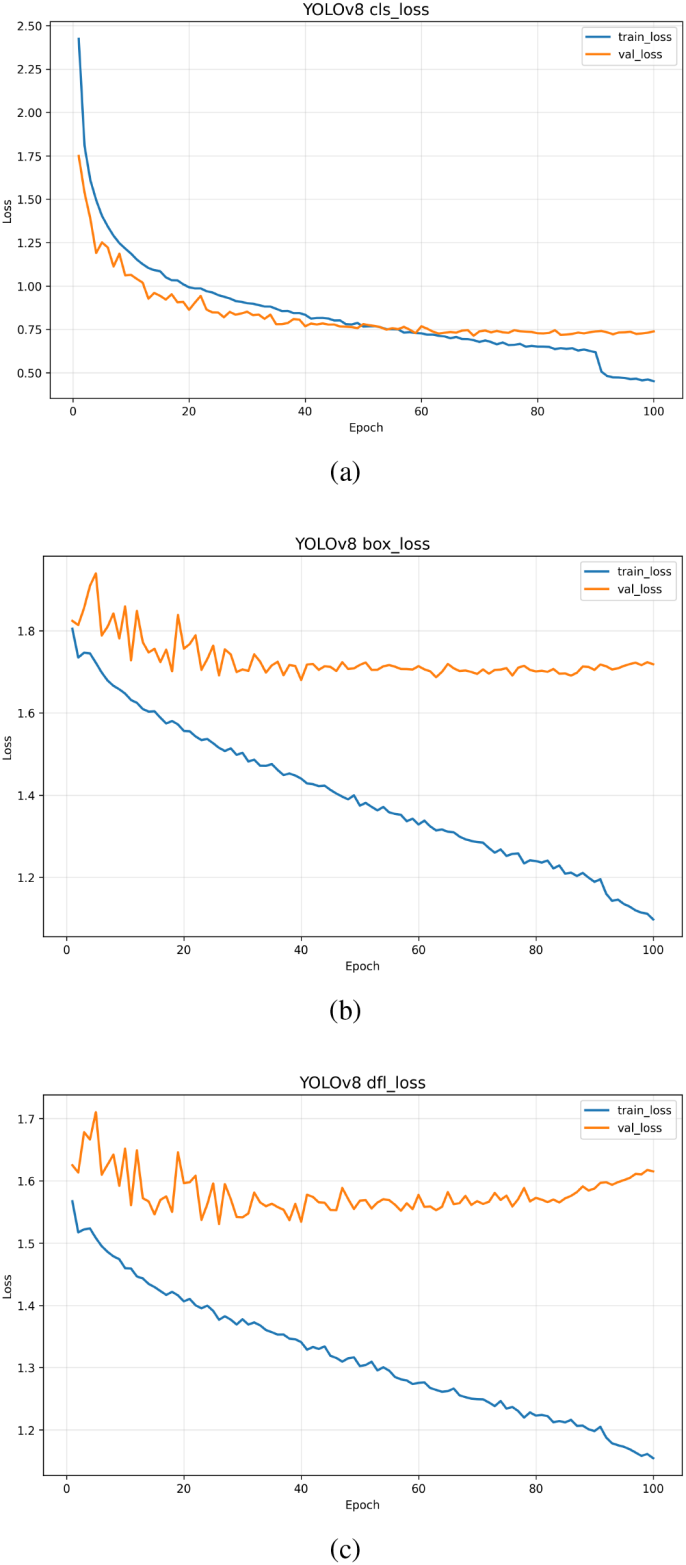
**(a)** Training and validation loss curves for the classification (cls) component of YOLOv8, showing rapid convergence and stable performance. **(b)** Box regression (box) loss over epochs, indicating consistent training progress with minor fluctuations in validation loss. **(c)** Detection loss for the detection (dfl) component, demonstrating steady decrease in both train and val losses, though with slight divergence in later epochs.

For YOLOv8, three loss components reveal nuanced performance: Classification loss (cls loss, **Figure 7a**) shows both training (blue) and validation (orange) losses starting high (2.40 vs. 1.75) and declining, with training loss dropping abruptly to 0.45 near epoch 100 to narrow the gap. Despite a slight persistent divergence post-epoch 50, overall alignment indicates robust generalization for classification tasks. Bounding box regression loss (box loss, **Figure 7b**) highlights training loss (blue) monotonically decreasing from 1.8 to 1.2, reflecting steady localization refinement, while validation loss (orange) fluctuates (1.6–1.1) but trends downward, suggesting acceptable generalization despite noise or scale diversity in the dataset. Notably, Distribution Focal Loss (dfl loss, **Figure 7c**) uncovers potential challenges: training loss (blue) smoothly declines from 1.55 to 1.15, yet validation loss (orange) oscillates wildly (1.5–1.7) and rises mildly post-epoch 70, hinting at overfitting or suboptimal learning rate settings.

Collectively, these analyses demonstrate Faster R-CNN’s stable convergence, SSD’s overfitting tendencies, and YOLOv8’s balanced performance across losses—with cls loss and box loss indicating strong generalization, while dfl loss raises minor concerns. This multi-faceted evaluation provides critical insights into model selection and optimization for tea garden weeding detection tasks. The lower performance mainly results from class imbalance and the small number of tool samples; we did not perform additional tuning as our goal is baseline benchmarking rather than model optimization.

### 5.3 Visualization results analysis

**Figure 8:**
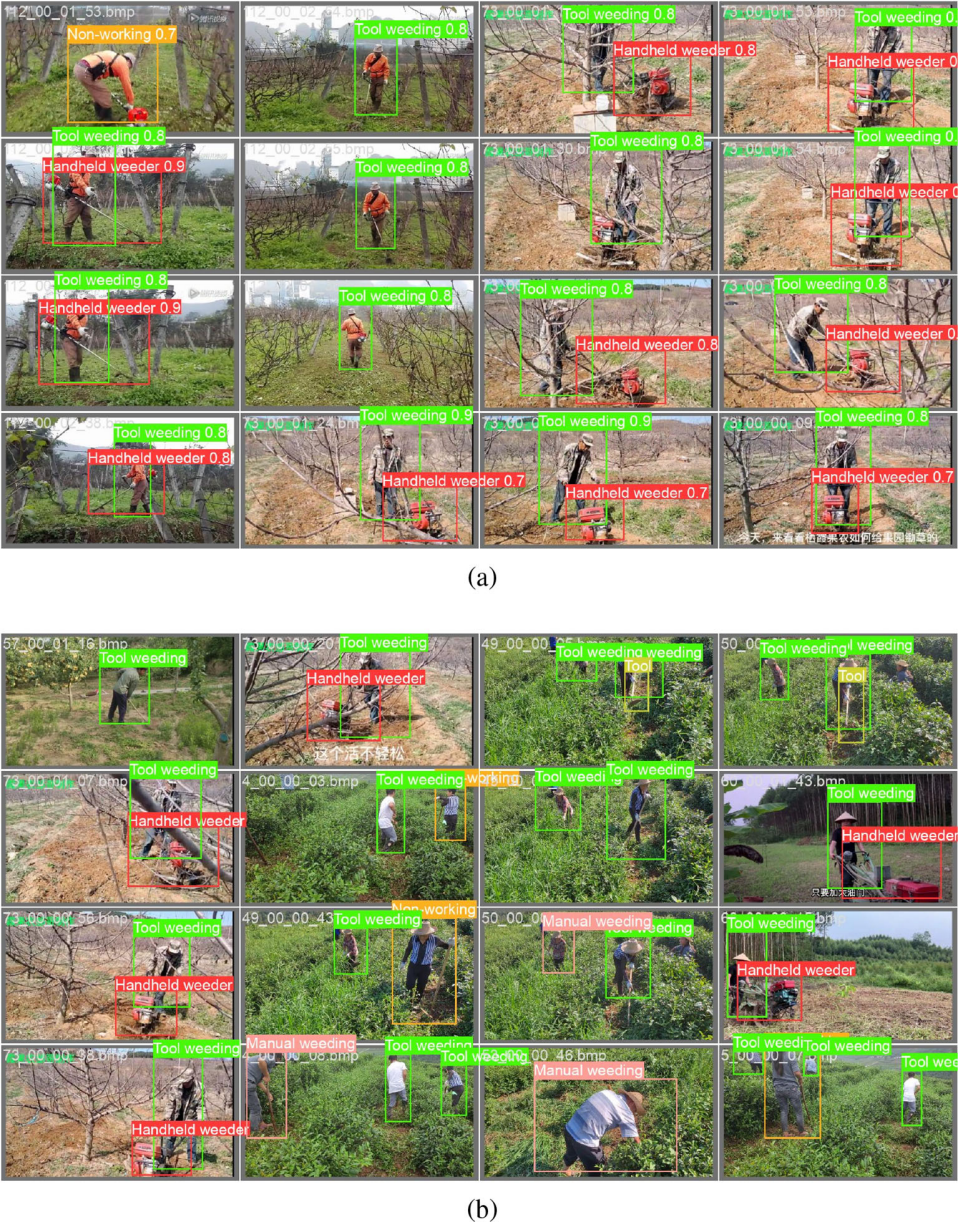
**(a)** Visualization of detection results on the test set using the proposed model, with bounding boxes and class labels overlaid; red boxes indicate false positives or misclassifications, while green boxes denote correct predictions. **(b)** Qualitative comparison of detection performance across diverse environmental conditions, including varying lighting, occlusion, and background clutter, demonstrating robustness in real-world agricultural scenarios.

For clarity, we report behavior recognition performance and tool object detection performance separately, as they represent two independent tasks. The **Figure 8** presents a comparison between the predictions generated by the YOLOv8 model and the corresponding ground-truth annotations on the test set. The visualization results demonstrate that YOLOv8 is capable of accurately detecting various weeding behaviors and associated tools across diverse scenarios, including manual weeding, tool-assisted weeding, and mechanical weeding. In well-lit conditions, the model exhibits particularly strong performance, with detection bounding boxes precisely localized and class assignments consistently correct.

Even in partially occluded or complex background scenes, YOLOv8 maintains robust detection performance. However, challenges remain in detecting small-scale targets, such as distant tools, where instances of missed detections or misclassifications occasionally occur. In scenarios where multiple individuals perform weeding simultaneously, the model is able to detect multiple targets concurrently, yet in regions with dense object distribution, overlapping or confused detection boxes can still arise.

Overall, these visualization results confirm that the tea garden weeding behavior recognition dataset constructed in this study supports effective training and evaluation of mainstream object detection algorithms. The dataset enables reliable detection across varying operational conditions, indicating its high quality and practical applicability. Among the models evaluated, Faster R-CNN demonstrates the best overall performance in terms of accuracy and robustness, YOLOv8 achieves notable advantages in precision and real-time detection capability, and SSD excels in computational efficiency.

While we present visualization results for YOLOv8 due to its balanced performance, qualitative observations from SSD and Faster R-CNN showed similar detection patterns, though with variations in localization precision and false positive rates as reflected in their quantitative metrics. These findings collectively validate both the effectiveness of the proposed dataset and the potential of deep learning-based object detection methods for automated monitoring of tea garden weeding activities.

## 6 Discussion

This work will lead to several research directions. Firstly, multimodal perception and data fusion represent a promising approach for further development. Although the current datasets mainly rely on RGB images, integrating infrared thermal imaging can enhance recognition capabilities in weak light conditions such as dawn and dusk, and combining with depth sensing will significantly improve the three-dimensional positioning of tools and human operations.

The dataset exhibits noticeable class imbalance, particularly between the “Tools” (155 samples) and “Machine Weeding” (1,536 samples) categories. Per-class evaluation results reveal that this imbalance contributes to the lower performance in the “Tools” class. We discuss this limitation and note that future work may incorporate re-sampling strategies or weighted loss functions to mitigate imbalance effects. Future data collection efforts could aim to balance the number of tool samples, and data augmentation techniques such as rotation, scaling, or synthetic image generation could be employed to enhance the diversity and quantity of tool instances. Although the dataset was designed with multi-view acquisition, this study does not include an ablation experiment comparing single-view and multi-view configurations. Therefore, the contribution of multi-view information is discussed as a potential advantage rather than a validated performance factor. Future work will include dedicated ablation experiments to quantify the benefit of each additional view.

Time modeling also offers important opportunities. Extending current static image analysis to video sequence understanding using LSTM or Transformer architectures will enable the capture of the continuity and dynamics of weeding actions, bridging the gap between behavior recognition and operation quality assessment. For categories with limited samples, such as tools and handheld lawn mowers, strategies like meta-learning or enhancements based on generative adversarial networks can alleviate detection bottlenecks.

In the long run, combining visualized data with agricultural knowledge graphs (including crop growth cycles, weed distribution patterns, and environmental factors) can achieve semantic-level fusion and build intelligent decision-making systems with domain awareness. This integration will promote the closed-loop “perception-analysis-decision-making” framework of precision agriculture.

In conclusion, this dataset provides a solid foundation for multi-perspective visual understanding of weeding behavior in tea gardens and offers a multi-functional platform for promoting the application of smart agriculture. Future research that utilizes multimodal data fusion, temporal behavior analysis, and domain-informed decision support has the potential to further expand the applicability of this dataset and deepen the implementation of smart agriculture in complex operational scenarios.

## 7 Conclusions

This study presents the construction and public release of a multi-view visual dataset for tea garden weeding behavior recognition, addressing a critical gap in agricultural behavior analysis where dedicated datasets for weeding activities are lacking. The dataset comprises 6,473 high-quality images, annotated across six categories: manual weeding, tool-assisted weeding, mechanical weeding, tools, handheld weeders, and non-working states. All samples were meticulously annotated and provided in both COCO and YOLO formats, enabling immediate use with mainstream object detection frameworks such as YOLOv8, SSD, and Faster R-CNN. In its current form, this work serves primarily as a dataset contribution, providing a valuable resource for future research on multimodal plant disease detection.

Data acquisition employed synchronized frontal, lateral, and top-down views, capturing complementary features of the same action from different perspectives. This multi-view design mitigates challenges arising from occlusion, posture variability, and background clutter in complex tea garden environments. The images were sourced from 108 video segments—53 captured *in situ* at Dangmenpo Tea Garden, Meziyuan Village, Pingli County, Ankang, Shaanxi Province, and 55 collected via web crawling—spanning diverse domestic and international tea garden scenarios and multiple times of day, thereby ensuring environmental diversity and lighting variation.

Experimental evaluations using Faster R-CNN, YOLOv8, and SSD on a standardized train-validation-test split indicate strong dataset utility and inherent challenges. Faster R-CNN achieved an mAP of 82.24%, while YOLOv8 attained the highest precision of 83.7%. These results demonstrate that the dataset effectively supports accurate detection of weeding behaviors and tools while remaining challenging for contemporary object detection models. Importantly, the dataset facilitates three-dimensional semantic representation of weeding actions, offering a unified platform for behavior understanding, tool identification, and operational state monitoring in complex agricultural settings. This provides direct support for applications including intelligent weeding robots, precision agricultural monitoring systems, and farm operation quality assessment, thereby contributing to the automation and efficiency enhancement of tea garden management while reducing labor costs and safeguarding the production of high-value crops.

## Data Availability

The datasets presented in this study can be found in online repositories. The names of the repository/repositories and accession number(s) can be found below: https://dx.doi.org/10.21227/eh4y-yv31.
